# Spatial amine metabolomics and histopathology reveal localized brain alterations in subacute traumatic brain injury and the underlying mechanism of herbal treatment

**DOI:** 10.1111/cns.14231

**Published:** 2023-05-14

**Authors:** En Hu, Tao Tang, You‐mei Li, Teng Li, Lin Zhu, Ruo‐qi Ding, Yao Wu, Qing Huang, Wei Zhang, Qian Wu, Yang Wang

**Affiliations:** ^1^ Department of Integrated Traditional Chinese and Western Medicine, Institute of Integrative Medicine Xiangya Hospital, Central South University Changsha Hunan China; ^2^ National Clinical Research Center for Geriatric Disorders Xiangya Hospital, Central South University Changsha Hunan China; ^3^ College of Chemistry and Chemical Engineering Central South University Changsha Hunan China; ^4^ Department of Neurology Xiangya Hospital, Central South University Changsha Hunan China; ^5^ The College of Integrated Traditional Chinese and Western Medicine Hunan University of Chinese Medicine Changsha Hunan China

**Keywords:** amine metabolites, angiogenesis, spatial metabolomics, traumatic brain injury, Xuefu Zhuyu decoction

## Abstract

**Introduction:**

Spatial changes of amine metabolites and histopathology of the whole brain help to reveal the mechanism of traumatic brain injury (TBI) and treatment.

**Methods:**

A newly developed liquid microjunction surface sampling–tandem mass tag–ultra performance liquid chromatography–mass spectrometry technique is applied to profile brain amine metabolites in five brain regions after impact‐induced TBI at the subacute stage. H&E, Nissl, and immunofluorescence staining are performed to spatially correlate microscopical changes to metabolic alterations. Then, bioinformatics, molecular docking, ELISA, western blot, and immunofluorescence are integrated to uncover the mechanism of Xuefu Zhuyu decoction (XFZYD) against TBI.

**Results:**

Besides the hippocampus and cortex, the thalamus, caudate‐putamen, and fiber tracts also show differentiated metabolic changes between the Sham and TBI groups. Fourteen amine metabolites (including isomers such as L‐leucine and L‐isoleucine) are significantly altered in specific regions. The metabolic changes are well matched with the degree of neuronal damage, glia activation, and neurorestoration. XFZYD reverses the dysregulation of several amine metabolites, such as hippocampal Lys‐Phe/Phe‐Lys and dopamine. Also, XFZYD enhances post‐TBI angiogenesis in the hippocampus and the thalamus.

**Conclusion:**

This study reveals the local amine‐metabolite and histological changes in the subacute stage of TBI. XFZYD may promote TBI recovery by normalizing amine metabolites and spatially promoting dopamine production and angiogenesis.

## Graphical abstract

The study uses an LMJSS‐TMT‐UPLC‐MS method to spatially map amine metabolites to brain histopathological changes including neuron loss, glial cell activation, and neurogenesis. This integrated study explains the mechanisms of posttraumatic brain injury brain damage and repair. It also uncovers the therapeutic mechanisms of traditional Chinese medicine, Xuefu Zhuyu decoction.

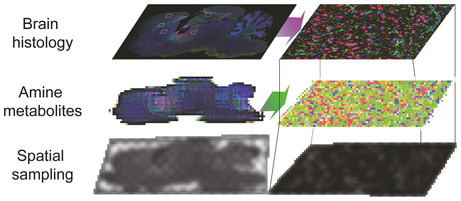

## 1 INTRODUCTION

Traumatic brain injury (TBI) is a major cause of global death and disability, especially for the moderate‐to‐severe subtypes. Despite therapies and medical care advances, most survivors suffer from multiple and prolonged sequelae.^1^ Thus, TBI is still a medical and socioeconomic challenge worldwide. The diverse sequelae primarily result from complex and spatial pathology all over the brain.^2^ However, previous studies mainly focus on the pathology and therapeutic response of the directly injured site, the cortex (CTX), and the nearby lesion, the hippocampus (HP),^3^, ^4^ distal changes, and the underlying mechanisms remain elusive.

Metabolomics of the biofluids or brain tissue is widely used to unveil the molecular mechanisms of TBI progression and therapeutic response.^4^, ^5^, ^6^, ^7^ From previous studies, the significantly changed metabolic pathways after TBI mainly involve amine metabolism, including amino acid metabolism, whose dynamics are pivotal for brain functioning.^4^, ^8^ For example, excessive glutamic acid in the brain leads to excitotoxicity; reduced γ‐aminobutyric acid (an inhibitory neurotransmitter) results in seizures.^9^ Besides, compromised branched‐chain amino acids implicate cognitive dysfunction after TBI.^10^, ^11^ Previous untargeted bulk‐metabolomics study has shown different metabolic alterations in the HP and CTX after TBI. For instance, the phenylalanine and proline were decreased in the CTX but not in the HP. However, few studies focused on the spatial changes of amine metabolites in brain regions other than the two regions aforementioned. In addition, the correlations between the spatial changes of metabolites and the histopathological differences are not investigated for a full explanation of the underlying mechanisms.

Mass Spectrometry Imaging (MSI) has been recently developed to spatially map the metabolites to tissue slices, which enables the exploration of in situ metabolite–histology correlations.^12^ However, current spatial‐resolved mass spectrometry methods cause a low detection coverage, low quantitation accuracy, and poor isomers—distinguish the ability for amino acids and other amine metabolites. Because it is difficult to spatial quantifies amino acids by spiking exogenous standards on tissue. It is also challenging to distinguish amine isomers, such as leucine/isoleucine and α‐alanine/β‐alanine, by Mass Spectrometry (MS).^12^

To address these limitations, we developed liquid microjunction surface sampling (LMJSS)–tandem mass tags labeling (TMT)–ultra‐performance liquid chromatography (UPLC)–MS technique.^12^ The LMJSS system uses a spot‐to‐spot sample to gain spatial information. It enables in‐site extraction of metabolites and allows the addition of exogenous standards precisely to each extract for accurate calibration and quantification.^13^, ^14^ Hyphenated LMJSS‐MS has been successfully performed to quantify the sphingolipids in brain tissues and to track the spatial changes of lipids in brain tissues after TBI and its treatment.^15^ TMT6 reagents label the primary amine of amine metabolites. It helps distinguish different samples in a pooled mixture, reducing the analysis time and sample‐to‐sample detection errors. Additionally, it improves the sensitivity and quantitative accuracy of amine metabolites.^16^ Moreover, the TMT labeling couples the UPLC system to discern isomers. Compared with the traditional methods, our new method provides quantitative spatial information on amino acids and other amine metabolites and leads to the differentiation of their isomers.^12^

Due to the complexity and regional heterogeneity of TBI pathology, polytherapies, rather than monotherapies, tend to achieve pleasing outcomes because of multitargets and synergistic effects.^17^ Xuefu Zhuyu decoction (XFZYD) is a traditional Chinese medical formula to treat cardiac‐cerebral vascular diseases associated with “blood stasis,” including TBI^6^, ^18^, ^19^; the underlying mechanisms are largely unknown. Previously, plasma metabolomics is used to evaluate the metabolic response of the whole body to XFZYD after TBI.^20^ As abnormalities within the blood may not be consistent with those in the local lesions, especially in the brain with the blood–brain barrier, metabolomics of brain tissues will help us find more direct and reliable in situ cues about the mechanism of XFZYD against TBI. Recently, we adopted untargeted metabolomics to detect the metabolomic changes in post‐TBI HP after XFZYD treatment. This study uncovers the different mechanisms of XFZYD to protect HP from injury and to facilitate brain repair in the acute and chronic stages of TBI, respectively.^6^ However, the metabolic responses in the subacute phase, when the effect of XFZYD shifts from limiting injury to promoting repair, remain unclear. Also, the changes in amine metabolites in the XFZYD‐treated TBI brain have not been quantified. Moreover, the metabolic regulations of XFZYD on the key brain regions other than the CTX and the HP are worth exploring to reveal the holistic therapeutic efficacy of XFZYD against TBI beyond local improvement.

The present study aims to quantitatively illustrate the spatial changes of amine metabolites (including isomers) and their relationship with the histopathology in five brain regions: the CTX, the HP, the thalamus (TH), the caudate‐putamen (CP), and the fiber tracts (FT) in the subacute stage of TBI, and to explore the mechanism of XFZYD against TBI based on the spatial metabolite–histopathology changes, and molecular biological investigation.

## 2 MATERIALS AND METHODS

### 2.1 Preparation and storage of XFZYD

XFZYD was prepared as we previously reported.^6^, ^15^ In brief, 11 dried herbs (Table S1) were mixed and extracted with double‐distilled water, vacuum‐lyophilized to powder (yield = 16.9%, w/w). The powder was partitioned, sealed in a vacuum bag, and stored at −80°C. The working solution was made at a final concentration in sterilized water shortly before use. The concentrations of four major components were quantified by LC‐MS (Figure S1 and Table S2).

### 2.2 Animal experiments

All animal protocols were approved by the Committee on the Use and Care of Animals of Central South University (NO. 201803421). Male Sprague Dawley (SD) rats (220–240 g, 8–9 weeks) were purchased from the Hunan Slake Jingda Laboratory Animal Co., Ltd. Rats were housed according to the Guide for the Care and Use of Laboratory Animals of the National Institutes of Health (NIH Publication No. 85–23, revised 1996) in the SPF condition.

Rats were randomly assigned into the Sham, controlled cortical impact (CCI), and XFZYD groups. The CCI model was adopted to replicate a severe TBI with the following parameters: impact depth, 5.0 mm; striking speed, 6.0 m/s; dwell time, 50 ms.^15^ The rats in the Sham group underwent the same surgery as CCI animals, except for the hit on the right CTX. The XFZYD group was intra‐gastrically administered with 1.52 g/kg XFZYD solution (corresponding to 9 g/kg crude herbs) once daily. This dose effectively treats TBI according to previous studies.^7^, ^21^, ^22^, ^23^ As controls, an equal volume of distilled water was given to the Sham and the CCI rats.

### 2.3 Sample collection and pretreatment

Rats were randomly assigned into three parts according to different aims: (1) *n* = 3 for Western Blotting and ELISA; (2) *n* = 3 for histological examination; (3) *n* = 5–8 for spatial metabolomics. Rats were killed by an overdose of sodium pentobarbital injection (100 mg/kg, i.p.). After perfusion with normal saline through the left ventricle, rat brains were removed. For spatial metabolomics, brains were snap‐frozen in liquid nitrogen and cut into 20‐μm‐thick sagittal sections. For histological and immunofluorescent detection, the brains were fixed with 4% paraformaldehyde, embedded in paraffin, and cut into 3‐μm‐thick sagittal sections. For western blot and ELISA analysis, the HP and the prefrontal CTX distal to the wound were isolated, snap‐frozen, and stored at −80°C separately.

### 2.4 In situ spatial resolved sampling and amine labeling

**FIGURE 1:**
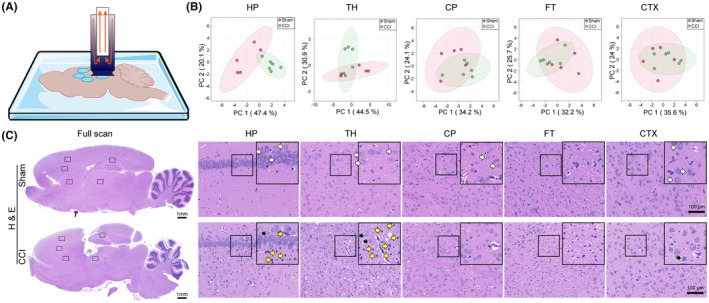
Spatial amine metabolic profile and histological changes in traumatic brain injury (TBI) brains. (A) LMJSS‐based spot‐to‐spot extraction. (B) PCA plots of amine metabolites (including amino acids) in the Sham and CCI groups. (C) H&E staining shows disorganized hierarchy, neuron pyknosis (black arrow), and inflammatory cell infiltration (yellow arrows) in the CCI brain. *n* = 5 in the Sham group, *n* = 6 in the CCI group (B); *n* = 3 (C).

An LMJSS system was employed for spatial‐resolved sampling based on our previous work^12^ (Figure 1A). In brief, the extraction solution (10% hexafluoroisopropanol‐40% methanol‐0.5% acetic acid‐water) was delivered through the outer capillary of the flow probe at a flowrate of 10 μL/min, which formed a Liquid micro junction on the probe–sample interface. Then, the extracts were aspirated through the inner capillary and collected into an injection loop (11 μL). X‐Y‐Z three linear stages controlled by a moving controller were built to fulfill spatial sampling. Five regions (HP, TH, caudate‐putamen (CP), fiber tracts (FT), and prefrontal CTX distal to the wound) were scanned by the probe spot by spot (six spots per region).

The extracts of each region were differentially isotopically labeled with 6‐plexed TMT6 reagents and then pooled. For amino acid quantification, 3 μL of 13 amino acids standards (5 μg/mL) was labeled with TMT0 and mixed with TMT6‐labeled brain extracts for LC‐MS analysis.

### 2.5 LC‐MS analysis

The UPLC system (LC‐30 AD, Shimadzu) was equipped with an ACQUITY UPLC HSS C18 (2.1*50 mm, 1.7 μm, Waters Corporation). The mobile phase A was 0.1% formic acid (v/v), and phase B was acetonitrile and formic acid (100:0.1, v/v). Separation was performed with a gradient from 5% to 10% B (0–9 min) and from 10% to 25% B (9–15 min) at a 0.4 mL/min flow rate. The mass spectrometer parameters were: full scan mode; m/z range: 50–500; multiple‐stage fragmentation scan modes; positive ion mode; capillary voltage: 4500 V; nebulizing gas (N_2_) rate: 1.5 L/min; collision energy: 50%. Other parameters were similar to our previous study.^12^ For identification, the significantly changed metabolite ions were selected for fragmentation, and the metabolites with the characteristic six reporters of TMT6 labels as fragments were identified as amine metabolites. Those amine metabolites were further identified by searching the MS or MS/MS features in the HMDB database (https://hmdb.ca/) and MSFinder (Table S3).

### 2.6 Histological examination

Paraffin sections were firstly deparaffined and rehydrated. For H&E staining, the rehydrated slices were stained with an H&E kit (G1004 and G1001, Servicebio). While Nissl staining was proceeded with toluidine blue solution (G1036, Servicebio). For immunofluorescence, the slices were incubated with primary antibodies overnight at 4°C, secondary antibodies for 1‐hour incubation at room temperature, and DAPI solution (C0065, Solarbio) for 5 min. The slices were scanned using a Pannoramic Midi Scanner (3D Histech) and processed with the Caseviewer 2.3 (3D Histech).

### 2.7 Western blot

Samples were incubated with rabbit anti‐Slc7a5 (BA3479, BOSTER) and rabbit anti‐Slc3a2 (1:250, bs‐6659R) antibodies. An HRP‐conjugated goat anti‐rabbit IgG antibody (111‐035‐003, Jackson ImmunoResearch) was used as the secondary antibody. ECL reagents were added to visualize the antigen–antibody intensity with a Gel Doc XR Imager (Bio‐Rad, Bio‐Rad Laboratories GmbH, Heidemannstrasse). The bands were quantified by Image Lab software (Bio‐Rad).

### 2.8 ELISA

A rat ELISA kit of dopamine (ZC‐36551, ZCIBIO Technology Co. Ltd) was utilized according to the manufacturer's instructions. Briefly, the brain tissue was homogenized in PBS at a ratio of 1:9 (w/v). Fifty microliters of supernatant was incubated with 100 μL HRP‐conjugated detecting antibody at 37°C for 1 h. Then, substrates were added at dark for 15‐min reaction. Eventually, the O.D. was read at 450 nm. The sample concentrations were calculated according to the standard curve.

### 2.9 Molecular docking

The 3D structures of tyrosine, Lys‐Phe, and Phe‐Lys were acquired from ZNIC15 (http://zinc.docking.org/; ZINC266964; ZINC2522657; ZINC13520785). The crystal structures of tyrosine hydroxylase were obtained from the RCSB Protein Data Bank (https://www.rcsb.org/, identifier: 1TOH). The docking process was calculated by the Genetic Algorithm. Other parameters were set as default. Finally, the molecular conformations of the ligands with the highest affinity to the targets were visualized by PyMoL.

### 2.10 Data processing and statistical analysis

The peak area, height, and retention time of amine metabolites from the LC‐MS dataset were extracted. Peak areas of amino acids were calibrated by their corresponding TMT0‐labeled amino acid standards. Other amine metabolites were calibrated by the total peak area of all the TMT0‐labeled amino acids as internal standards. Data were imputed into metaboAnalyst 5.0 (https://www.metaboanalyst.ca/) for Principle Component Analysis (PCA) and heatmap visualization after log transformation and Pareto scaling. The STITCH website constructed the metabolite–protein network (http://stitch.embl.de/), in which Rattus norvegicus was selected as the resulting species.

Statistics were done by *SPSS* (version 26, IBM Corp.). For the high‐throughput metabolomics data, the Student's *t*‐test was used. For the rest, normal distribution was checked by the Shapiro–Wilk test. Then, the Student's *t*‐test was applied to the normally distributed data. Otherwise, the Mann–Whitney U test was adopted. One‐way ANOVA was adopted for the three‐group comparison. *p* < 0.05 was considered statistically significant.

## 3 RESULTS

### 3.1 Spatial changes of amine–metabolite profile in rat brains after severe TBI by LMJSS‐TMT‐LC–MS and their association with general histological alterations

Based on the LMJSS‐TMT‐LC‐MS method (Figure 1A), 13 amino acids could be quantified, including three pairs of isomeric amino acids differentiated (α‐alanine and β‐alanine, L‐leucine, and L‐isoleucine, L‐valine, and norvaline). Besides targeted amino acids, up to 500 additional untargeted LC‐MS features were detected. Among them, 26 features were significantly changed after TBI in at least one region, and six were identified as amine metabolites (Table S3). Spatio‐chemical differences based on the aforementioned amine metabolites between the Sham and CCI group of rats were explained by PCA. From the score plots, the metabolites profile of the samples in the CCI group was separated from that of the Sham in varying degrees over different brain regions (Figure 1B). The most apparent separation occurred in the HP.

H&E staining showed that on the 14th day after TBI, the brain defect penetrated the CTX and HP into the TH. The cells were disorganized with a looser extracellular matrix and soma atrophy. The nuclei of neurons showed pyknosis (black arrows), and inflammatory cells were under infiltration (yellow arrows). Among the five regions of interest, the TH and HP presented the most apparent histological changes (Figure 1C).

### 3.2 The significant changes of amine metabolites in different regions of rat brain after severe TBI by LMJSS‐TMT‐LC–MS

**FIGURE 2:**
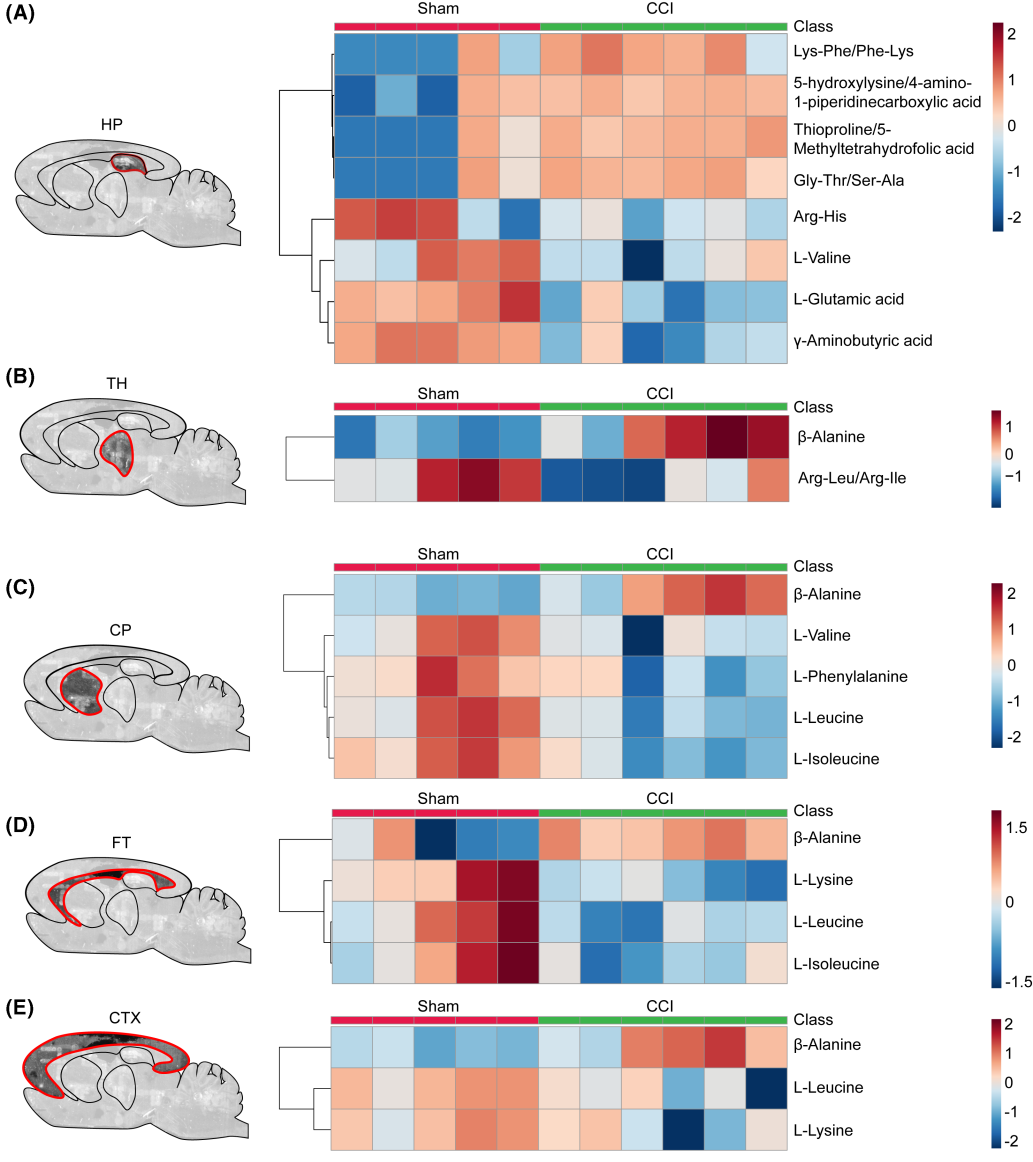
Spatial amine–metabolite changes after traumatic brain injury (TBI). (A) Heatmap of significantly expressed metabolites in the HP. (B) Heatmap of significantly expressed metabolites in the TH; (C) Heatmap of significantly expressed metabolites in the CP. (D) Heatmap of significantly expressed metabolites in the FT. (E) Heatmap of significantly expressed metabolites in the CTX. HP, hippocampus; TH, the thalamus; CP, the caudate‐putamen; FT, the fiber tracts; CTX, the cortex. Data are expressed as the mean ± SD; *n* = 5 in the Sham group; *n* = 6 in the CCI group.

For metabolites, eight metabolites were significantly altered (thresholds: *p* < 0.05 and FC >1.5 or <0.67) after TBI in the HP. Among them, Lys‐Phe/Phe‐Lys, 5‐hydroxylysine/4‐amino‐1‐piperidine carboxylic acid, thioproline/5‐methyltetrahydrofolic acid, and Gly‐Thr/Ser‐Ala were increased, while Arg‐His, L‐valine, L‐glutamic acid, and γ‐aminobutyric acid were decreased in CCI brains (Figure 2A). In the TH, β‐alanine was upregulated, while Arg‐Leu/Arg‐Ile was downregulated (Figure 2B). Of note, β‐alanine was also elevated significantly in the CP, FT, and CTX in CCI brains (Figure 2C‐E). Meanwhile, L‐valine, L‐phenylalanine, L‐leucine, and L‐isoleucine markedly declined in the CP of CCI sections (Figure 2C). In the FT, L‐lysine, leucine, and isoleucine were lowed after TBI (Figure 2D). Moreover, L‐lysine and L‐leucine also declined in the CTX (Figure 2E).

### 3.3 Histopathological alterations of rat brains in severe TBI

**FIGURE 3:**
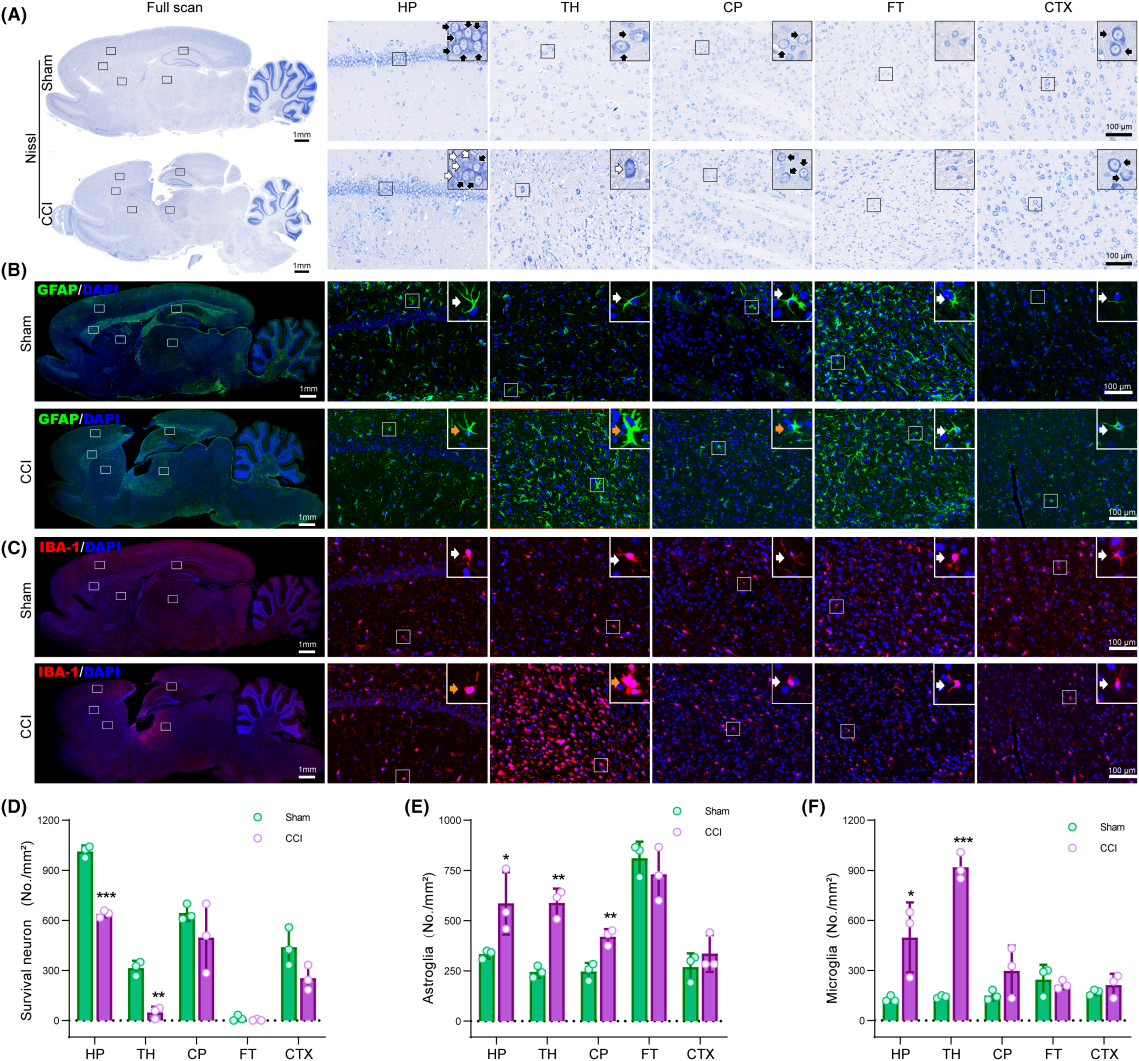
Spatial changes of neurons, astrocytes, and microglia in traumatic brain injury (TBI) brains. (A) Nissl staining visualizes survival neurons (black arrows) and injured neurons (white arrows). (B) Immunofluorescent staining of GFAP (green, astrocyte marker) shows rest astrocytes (white arrows) and active astrocytes (orange arrows). (C) Immunofluorescent staining of Iba‐1 (red, microglia marker) displays rest microglia (white arrows) and active microglia (orange arrows). (D) Statistic graph of survival neuron numbers in different brain regions. (E) Sstatistic graph of astrocyte counts in the brain. (F) Statistic graph of microglia numbers in the brain. HP, hippocampus; TH, the thalamus; CP, the caudate‐putamen; FT, the fiber tracts; CTX, the cortex. Data are expressed as the mean ± SD; *n* = 3; * *p* < 0.05; **p < 0.01 (compared with the CCI group).

Nissl staining displayed that the survival neurons were characterized by large cell bodies with light‐stained nuclei, prominent nucleoli, and Nissl bodies–enriched homogeneous cytoplasm (Figure 3A, black arrow). In comparison, the dying neurons showed decreased or blurred Nissl body and pyknotic nuclei (white arrow). In CCI brains, the neurons were almost all damaged in the TH while arranged disorderly in the HP and CTX. Statistically, survival neurons significantly decreased in the HP and TH regions after TBI (Figure 3A,D).

In Sham rats, most astrocytes and microglia were rest (Figure 3B,C, white arrows). Following TBI, both glial cells were spatially differentially activated and proliferated (Figure 3B,C,E,F). The number of astrocytes and microglia significantly increased in the TH and HP. Astrocytes were activated and induced hypertrophy in these regions (Figure 3B, orange arrow). Similarly, microglia were activated with shorter processes and enlarged soma (Figure 3C, orange arrow). However, the number and morphology of astrocytes and microglia did not change obviously in the FT and the distal CTX. In the CP, astrocytes proliferated and altered in cell morphology, but microglia did not.

**FIGURE 4:**
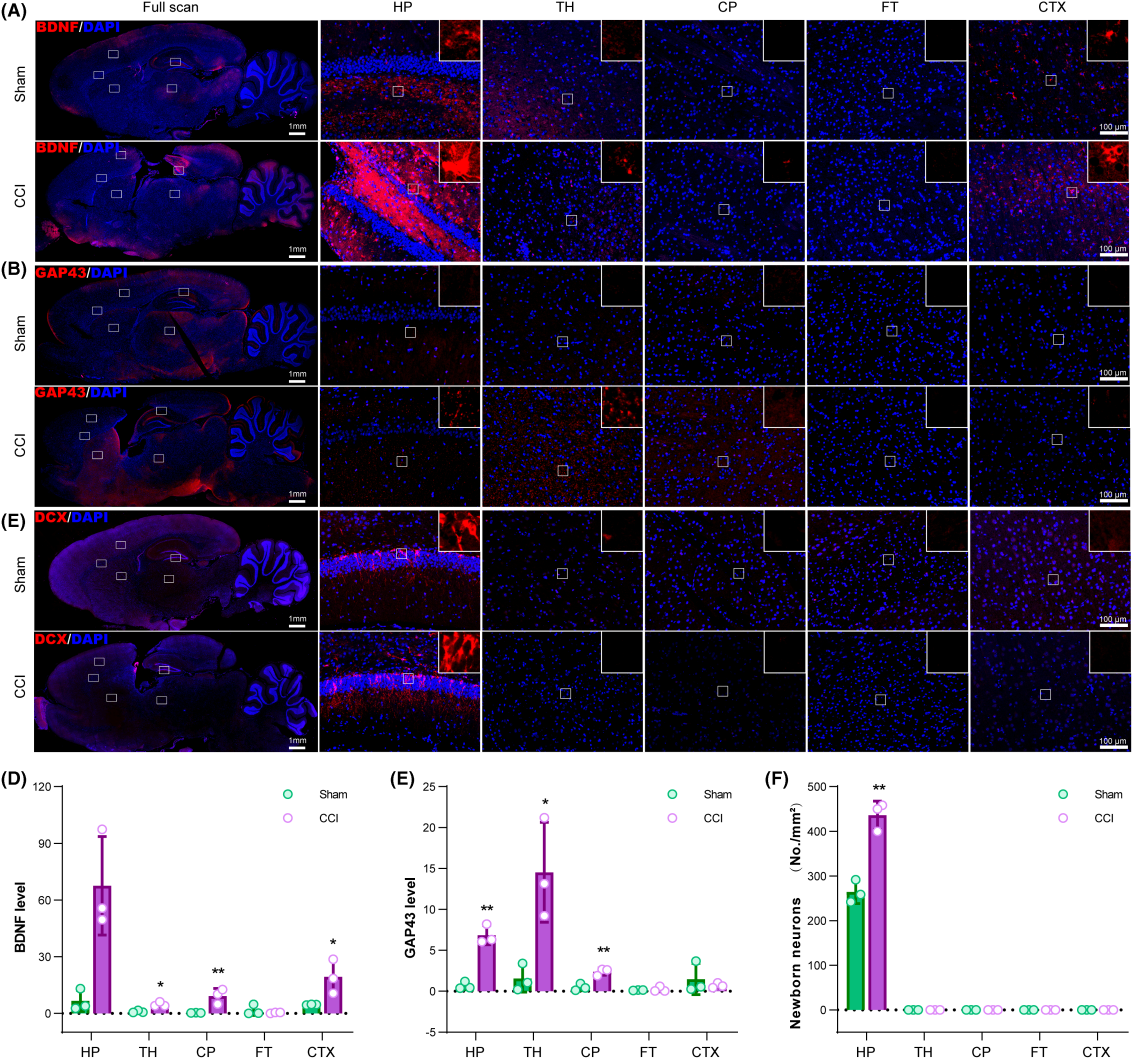
Spatial differences in BDNF, GAP43, and DCX in traumatic brain injury (TBI) brains. (A) Immunofluorescent staining of BDNF (red). (B) Immunofluorescent staining of GAP43 (Red). (C) Immunofluorescent staining of DCX. (D) Statistic graph of BDNF level in different brain regions. (E) Statistic graph of GAP43 density in the brain. (F) Statistic graph of DCX‐positive immature neurons in the brain. HP, hippocampus; TH, the thalamus; CP, the caudate‐putamen; FT, the fiber tracts; CTX, the cortex. Data are expressed as the mean ± SD; *n* = 3; * *p* < 0.05; **p < 0.01 (compared with the CCI group).

In the Sham group, BDNF is primarily expressed in the HP and the CTX. After TBI, BDNF levels in the HP, CTX, CP, and TH were largely elevated (Figure 4A,D). As for GAP43, the driver of axon growth, it was also significantly upregulated in the HP, CP, and TH (Figure 4B,E). Besides, DCX, the immature neuron marker, was only observed in the HP of both Sham and post‐TBI brains. Like BDNF and GAP43, DCX‐positive neurons increased in the HP after TBI (Figure 4C,F).

### 3.4 Effect of XFZYD on the significantly changed amine metabolites after severe TBI

**FIGURE 5:**
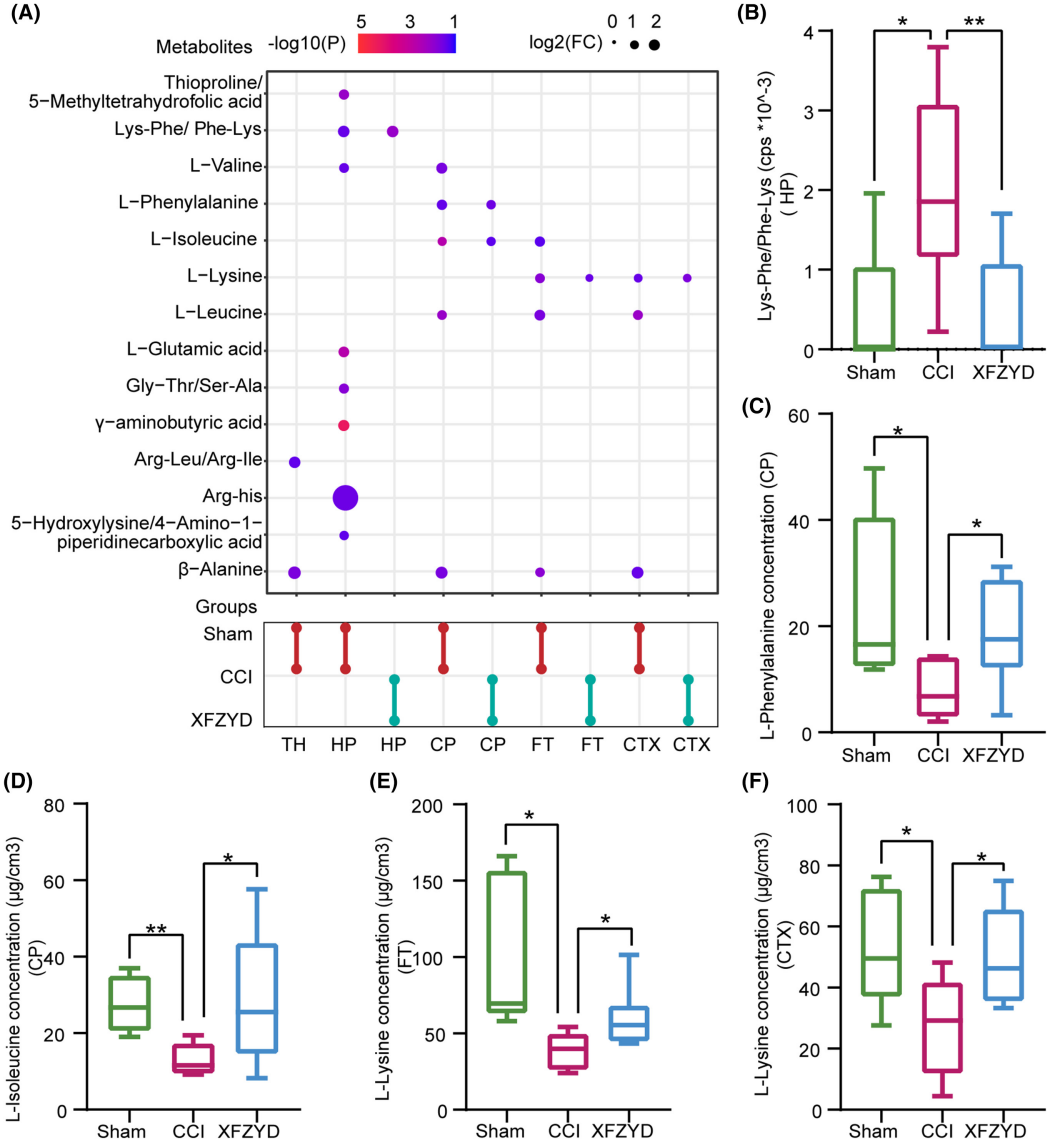
Spatially altered amine metabolites after XFZYD treatment after traumatic brain injury (TBI). (A) Significant expressed amine metabolites in the HP, the TH, the CP, the FT, and the CTX among the Sham, CCI, and XFZYD groups. The color gradient represented log2(FC); the redder, the more significant; dot size mapped ‐log10(*P*). (B) Relative abundance of Lys‐Phe/Phe‐Lys in the HP. (C) Concentrations of L‐phenylalanine in the CP. (D) The concentrations of L‐isoleucine in the CP. (E) The concentrations of L‐Lysine in the FT. (F) The concentrations of L‐Lysine in the CTX. HP, hippocampus; TH, the thalamus; CP, the caudate‐putamen; FT, the fiber tracts; CTX, the cortex.; *n* = 5 in Sham group, *n* = 6 in CCI group, *n* = 8 in XFZYD group; *p < 0.05; **p < 0.01 (compared with the CCI group).

PCA plots showed moderate differences between the CCI and XFZYD groups in the five regions (Figure S2A). It was found that XFZYD treatment significantly reversed the abnormality of Lys‐Phe/Phe‐Lys in the HP, L‐phenylalanine, and L‐isoleucine in the CP, as well as L‐lysine in the FT and the CTX (Figure 5).

### 3.5 Effect of XFZYD on brain histopathology after severe TBI

H&E staining indicated that XFZYD‐treated rats showed a more organized and dense extracellular matrix and displayed less soma atrophy, pyknosis, and inflammation, especially in the HP and the TH (Figure 1C and Figure S2B). Nissl staining suggested increased survival neurons in the TH and the CTX regions of XFZYD‐treated brains (Figure 3A and Figure S2B,G). Immunofluorescence showed insignificantly changed microglia status and numbers between the CCI and XFZYD groups (Figure 3B and Figure S2C,H). Astrocytes were markedly reduced in the CP after XFZYD administration (Figure 3D and Figure S2D,I).

### 3.6 Effect and mechanism of XFZYD‐responsive Lys‐Phe on severe TBI

**FIGURE 6:**
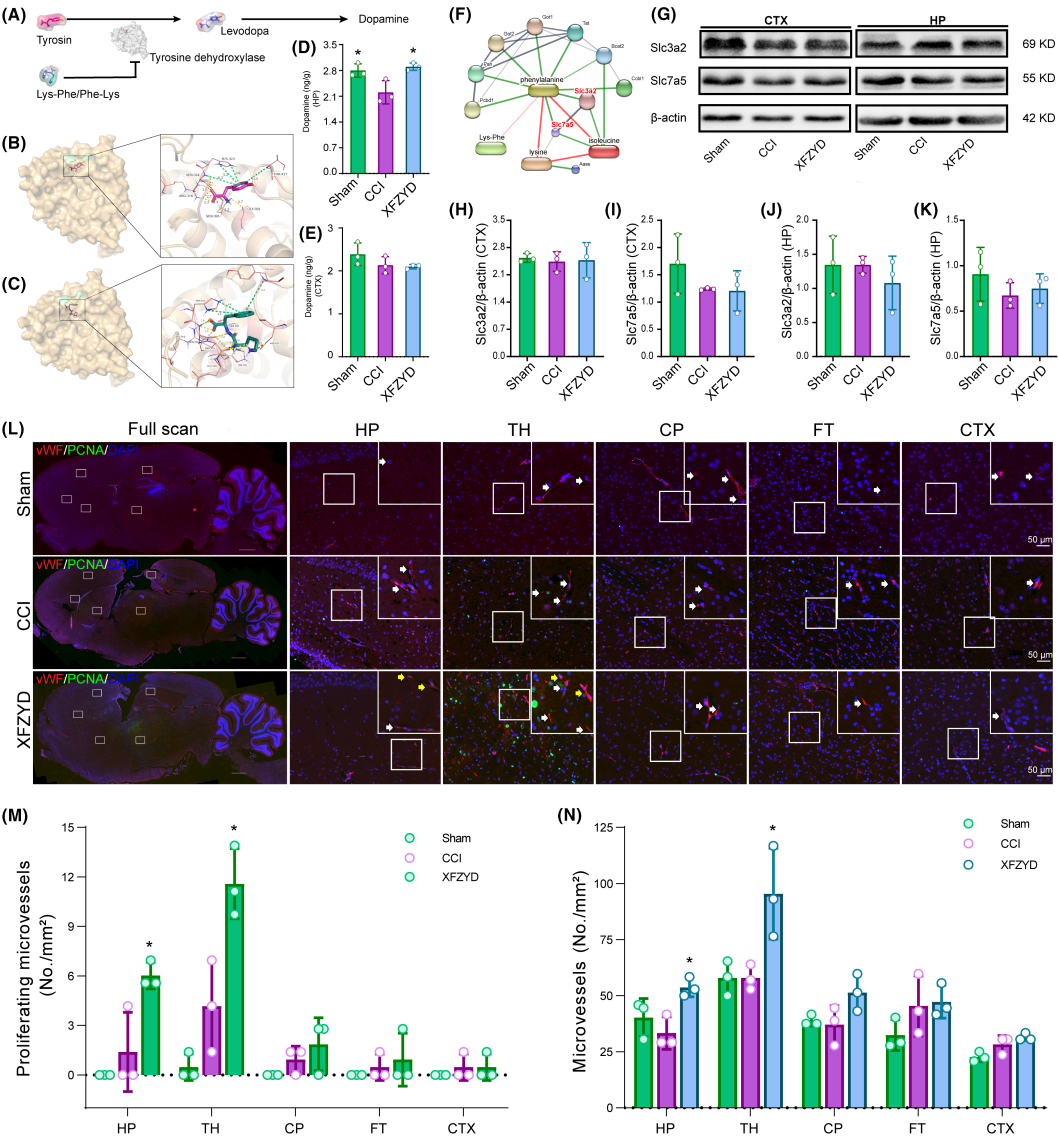
Mechanism of XFZYD on TBI recovery. (A) Putative mechanism of Lys‐Phe/Phe‐Lys on dopamine production. (B) Molecular docking of tyrosine hydroxylase with its natural substrate tyrosine. Yellow dotted lines: H‐bond; green dotted lines: pi‐cation bonds. (C) Molecular docking of tyrosine hydroxylase with Lys‐Phe anchored into the same pocket with tyrosine hydroxylase as tyrosine did. (D) XFZYD elevates dopamine concentration in the post‐TBI HP. (E) ELISA shows unchanged dopamine concentrations after TBI nor XFZYD treatment. (F) Metabolite‐protein network. Long oval, XFZYD altered metabolites; circle, metabolite associated proteins. (G) Representative western blot images of Slc3a2 and Slc7a5 in the CTX and the HP. (H‐I) Slc3a2 and Slc7a5 were not changed after TBI or XFZYD treatment in the CTX. (J‐K) Slc3a2 and Slc7a5 were not changed after TBI or XFZYD treatment in the HP. (L) Representative immunofluorescent images of vessels and proliferating cells. Red vessels; green, proliferating nucleus. (M) The number of proliferating vessels is significantly increased in the HP and TH regions after XFZYD treatment. (N) Vessel numbers are significantly increased in the HP and TH regions in the XFZYD group. HP: hippocampus; TH: thalamus; CP: caudate‐putamen; FT: fiber tracts; CTX: cortex; vWF: von Willebrand factor, endothelium marker; PCNA: proliferating cell nuclear antigen, proliferating marker. Data are expressed as the mean ± SD; *n* = 3; * *p* < 0.05 (compared with the CCI group).

Tyrosine hydroxylase is the rate‐limiting enzyme of dopamine biosynthesis^24^ (Figure 6A). Molecular docking indicated that tyrosine, the natural substrate of tyrosine hydroxylase, was embedded into the active pocket. The calculated binding energy was −6.5 kcal/mol. Tyrosine potentially formed seven hydrogen bonds (Figure 6B, yellow dot lines) and four pi–cation bonds (Figure 6B, green dotted lines) with tyrosine hydroxylase. Lys‐Phe was predicted to anchor into the same pocket as tyrosine (Figure 6C). The binding energy was −7.6 kcal/mol, lower than tyrosine. As measured, the Lys‐Phe probably interacted with tyrosine hydroxylase through 13 hydrogen bonds and three pi–cation bonds (Figure 6C). Meantime, the Phe‐Lys also interacted with tyrosine hydroxylase through seven hydrogen bonds and four pi–cation bonds. Their docking energy was −7.0 kcal/mol, also lower than that of the tyrosine–tyrosine hydroxylase complex (Figure S3).

ELISA indicated that TBI markedly reduced the dopamine level in the HP, while XFZYD reversed the level. In contrast, in the CTX, the level of dopamine was lower than that of the HP, but this alteration was not observed after TBI or XFZYD supplement (Figure 6D,E).

### 3.7 Spatial changes of vasculatures and transporters in rat brain related to the XFZYD‐responsive metabolites

The metabolite–protein interacting network demonstrated that two transporters of amino acids, the solute carrier family 7 member 5 (Slc7a5) and solute carrier family 3 member 2 (Slc3a2), were the core proteins that linked to the XFZYD‐changed metabolites (Figure 6F). Western blot suggested that CCI did not markedly change the levels of Slc3a2 and Slc7a5 in the HP and CTX, and neither did XFZYD (Figure 6G‐K and Figures S4‐S5).

As suggested by immunofluorescence, XFZYD increased the number of vWF^+^ vessels (red) in the HP and the TH after TBI (Figure 6L,M). Moreover, proliferating vessels (PCNA^+^ nucleus colocalized with vWF^+^ vessels) were also observed in the HP and TH of the treated brains (Figure 6L,N).

## 4 DISCUSSION

For the first time, we applied an LMJSS‐TMT‐UPLC‐MS‐based in situ amine metabolomics to illustrate the region‐specific alterations in the TBI and the XFZYD‐treated rat brains at subacute stages. The metabolic and histopathological changes after TBI are not only limited to the CTX and HP nearby the wound but also spread to the distal CTX, the CP, and the FT to different degrees. The most apparent amine–metabolite alterations occurred in the HP, including decreased L‐glutamic acid and GABA. At the same time, histology changed significantly in the HP and the TH. XFZYD partially reversed the abnormal amine metabolism. The subsequent experiments based on the XFZYD‐responsive amine metabolites indicated that XFZYD facilitates TBI recovery by promoting angiogenesis and increasing dopamine production.

### 4.1 Histological alterations after severe TBI and the underlying correlations to amine–metabolite changes

Microglia is the brain's primary inflammatory cell that may contribute to neurodegenerative and neurological deficits after TBI.^25^, ^26^ It interacts with astrocytes to mediate persistent neuroinflammation.^26^ In our study, the numbers of microglia and astrocyte were significantly increased in the HP and TH., accompanied by decreased survival neurons in these regions. It suggests the detrimental roles of microglia and astrocyte‐induced inflammation. Microglia can induce amine–metabolite changes. Since microglia is a debris scavenger in the central nervous system,^27^ it enzymatically degrades proteins to produce peptides and other amine waste in the necrotic brain regions. Therefore, the dipeptides such as Lys‐Phe/Phe‐Lys and Gly‐Thr/Ser‐Ala were upregulated after TBI in our study. In turn, serveral amine metabolites can also regulate microglia dynamics. For example, β‐alanine exposure activates microglia and promotes the secretion of pro‐inflammatory factors.^28^ In addition, the elimination of β‐alanine alleviates microglia‐induced neuropathic pain.^29^ The increased β‐alanine in the TH may participate in microglia activation and neuroinflammation after TBI.

HP shows more notable TBI‐associated histological and amine–metabolite alterations than those in the FT, the CP, and the CTX distal to the wound.^30^ It is consistent with previous research, indicating more severe damage nearby the injury than in the remote regions.^31^, ^32^ Three potential reasons may contribute to the significant discrepancy. First, HP is closer to the injury site than other regions. Therefore, it suffers more severe damage, resulting in more evident neuron loss, inflammatory cell proliferation, and the high degrading products of cell debris, Lys‐Phe/Phe‐Lys and Gly‐Thr/Ser‐Ala. Second, neurons are susceptible to insults such as mechanical force, excessive oxidative stress, and inflammation.^33^ Thus, the neuron‐enriched HP undergoes more significant neuron loss. As neurons are producers of neurotransmitters,^34^ L‐glutamic acid and γ‐aminobutyric acid are significantly diminished in the HP in our present study. Third, among the five regions, HP is the only one where the neural stem cells reside and proliferate to generate newborn neurons.^35^ Neurogenesis relies on metabolites, including amine metabolites. For instance, 5‐methyltetrahydrofolic acid, the active form of folic acid, is fundamental for neural development. Supplemental 5‐methyltetrahydrofolic acid protected newborns from neural tube defects.^36^, ^37^ Thereby, the upregulated 5‐methyltetrahydrofolic acid in HP may aid neurogenesis. Moreover, immature neurons utilize glutamine to fuel the tricarboxylic acid cycle.^38^ Therefore, the L‐glutamic acid is compromised in response to the enhanced neurogenesis in HP.

TH underwent similar histological changes with the HP, including decreased survival neurons, increased active astrocytes and microglia, and elevated BDNF and GAP43 expression. However, differential amine metabolites in the post‐TBI TH were less than in the HP. As discussed earlier, the fewer metabolic alterations in TH than in HP may come from the fewer neurons and the absence of neurogenesis. The histological changes in the TH regions were much more notable than those in most other studies.^39^, ^40^ Because we selected a relatively higher impact depth at 5 mm to simulate extremely severe TBI in the clinic. The direct injury penetrates the HP into the TH regions at this depth. Besides the initial insults, the histological changes in TH may also be caused by the altered amine metabolites. As our study shows, β‐alanine is significantly elevated in the TH. Previous studies have reported that β‐alanine can activate microglia to promote neuroinflammation.^28^, ^29^ Additionally, supplementary β‐alanine can upregulate BDNF in the brain.^41^ The upregulated β‐alanine agrees with the augmented microglia population and BDNF level in the TH.

### 4.2 The disturbance of amine metabolism in different brain regions during severe TBI and the underlying mechanisms

Most significantly changed amino acids were downregulated after TBI in the five areas of interest. It agreed with the results of previous bulk metabolomics in the HP, the CTX, the plasma, and the serum of TBI rats or pigs at acute stages.^4^, ^8^, ^42^ We assume it is caused by the enhanced decomposition of protein and amino acids, as evidenced by the elevated dipeptides and 5‐methyltetrahydrofolate, which carries one‐carbon units from catalyzed amino acids acids.^36^, ^37^ Spatially, the differentially expressed amine metabolites were mainly enriched in the HP, concordant with the significantly altered neurons, astrocytes, microglia populations, neurogenesis, and axon growth in our study.

The pathways related to significantly changed metabolites include glutamate and glutamic acid metabolism, L‐leucine, L‐isoleucine, and L‐valine degradation, and β‐alanine metabolism.

L‐glutamic acid and γ‐aminobutyric acid (GABA) are functionally antagonistic neurotransmitters in the brain. While releasing L‐glutamic acid from synapse triggers excitation, GABA induces inhibitory neurotransmission.^43^ Both are produced in neurons.^44^ In our study, L‐glutamic acid and GABA were explicitly decreased in the HP; it may be an integrated effect of diminished neuron amount, proliferated astrocytes, impaired energy production, and compromised L‐valine level. Firstly, L‐glutamic acid is released from the presynaptic neurons and transported into astrocytes. Then it converts to glutamine and transfers to neurons for L‐glutamic acid reproduction.^45^ L‐glutamic acid in the neuron is subsequentially degraded by glutamate decarboxylase to generate GABA in an energy‐dependent manner.^44^ As shown in our results, survival neurons were remarkably decreased. At the same time, astrocytes were increased in the HP, which means that L‐glutamic acid is mainly transferred into glutamine in the astrocytes and remains at a low level in the residual neurons. Secondly, compromised tissue perfusion and injured cellular energy metabolism reduce L‐glutamic acid and GABA.^44^ Consistent with the previous studies,^46^, ^47^ L‐glutamic acid and GABA were synchronically diminished with focal neuronal loss in the HP after TBI at the subacute stage. Thirdly, cytotoxic and ionic swelling astrocytes and neurons cause the unusual transport of vital glutamate and GABA.^44^ Lastly, L‐valine acts as an amino group nitrogen donor to synthesize glutamic acid.^48^ Our present study indicated that it agreed with the co‐declined L‐valine and L‐glutamic acid levels in the HP.

L‐leucine, L‐isoleucine, and L‐valine are BCAAs that are essential for brain metastasis.^11^ BCAAs can be catabolized to succinyl CoA or acetyl CoA, entering the tricarboxylic acid (TCA) cycle for energy production.^49^ During the recovery stages of TBI, more energy is needed in the affected brain regions for debris clearance, neurogenesis, and axonal and synaptic remodeling,^50^, ^51^ as indicated by the increased phagocytic microglia, neurotrophic astroglia, newborn neurons, and BDNF and GAP43 expression in our study. Thus, BCAA concentrations were decreased in most of the detected regions due to their catalyzation for energy production. It agrees with previous clinical studies indicating that BCAAs decrease the plasma of TBI patients, and supplemental BCAAs facilitate TBI recovery.^11^, ^49^

β‐alanine is an isomer of α‐alanine. In contrast to the proteogenic function of α‐alanine, β‐alanine in the central nervous system acts as a regulator factor.^41^ It can increase BDNF expression,^41^ which promotes neuronal survival, axonal sprouting, and synaptogenesis.^52^, ^53^ Supplementary β‐alanine increases brain BDNF, improves cognition, and mitigates symptoms of anxiety and depression.^41^, ^54^ It also prevented posttraumatic stress disorder (PTSD) in a rat model of mild TBI.^55^ In the present study, β‐alanine was upregulated in most regions of TBI brains, but α‐alanine did not change. The increased β‐alanine agrees with the elevated expression of BDNF and GAP43 in the affected areas. Moreover, as discussed earlier, β‐alanine can also trigger microglia activation and neuroinflammation.^28^, ^29^ Therefore, the exact effect of β‐alanine on post‐TBI pathophysiology needs further verification.

### 4.3 The therapeutic mechanism: XFZYD reverses amine metabolites and neurotransmitter dysregulation after severe TBI

Among the significantly changed metabolites after XFZYD treatment, only Lys‐Phe/Phe‐Lys and three essential amino acids (L‐phenylalanine, L‐lysine, and L‐isoleucine) were involved in TBI, so we focused on their changes. Earlier studies have demonstrated that XFZYD could reverse the abnormal lipids and metabolites in the post‐TBI rat brains and plasma.^6^, ^7^, ^8^ Similarly, the present spatial metabolomics implied that Xuefu Zhuyu decoction overturned the region‐specific dysregulation of three essential amino acids and a dipeptide.

L‐phenylalanine is a precursor for catecholamine synthesis, including three key neurotransmitters, norepinephrine, adrenalin, and dopamine. As mentioned earlier, they catabolized products of L‐isoleucine fuel for energy supplements. In this study, L‐phenylalanine and L‐isoleucine were significantly decreased in the CP but recovered after XFZYD treatment. Since the CP impairment primarily results in motor deficits,^56^ we speculate that XFZYD‐promoted motor improvements are partly contributed by the normalized L‐phenylalanine and the subsequential catecholamine synthesis, the recovered L‐isoleucine, and energy metabolism. L‐lysine can be catabolized to form L‐glutamic acid,^57^ regulating the balance of excitation/inhibition. Therefore, the XFZYD upregulated L‐lysine in the CTX, and the FT may restore the excitatory of the injured neurons by upregulating L‐glutamic acid.

Lys‐Phe/Phe‐Lys is a dipeptide that consists of lysine and phenylalanine residues in different orders. Poly‐Lys‐Phe can inhibit tyrosine hydroxylase activity,^24^ the rate‐limiting enzyme of dopamine production. In our study, molecular docking suggested that the dipeptides could bind with the active site of tyrosine hydroxylase. Their binding affinity was higher than that of tyrosine, the natural substrate. Besides, dopamine was negatively correlated with Lys‐Phe/Phe‐Lys in the HP after TBI and XFZYD treatment. Dopamine and its related signaling pathway are important in modulating cognition and memory.^58^, ^59^ Immediately after TBI, dopamine releases dramatically, inducing oxidative stress and neuroinflammation. In chronic conditions, dopamine decreases, resulting in dysfunction in long‐term depression, long‐term potentiation, and compromised memory, attention, and executive functions.^60^ In the HP, we detected declined dopamine on the 14th day after TBI. It can be classically interpreted by the loss of dopaminergic neurons in the brain.^58^, ^60^ Additionally, it provides an underlying explanation for dopamine regulation after TBI and XFZYD treatment: increased Lys‐Phe/Phe‐Lys inhibits the activity of tyrosine hydroxylase, by which to downregulate the level of dopamine post‐TBI. XFZYD pharmacologically decreases Lys‐Phe/Phe‐Lys and then ameliorates dopamine expression.

### 4.4 The therapeutic mechanism: XFZYD facilitates angiogenesis after severe TBI

Essential amino acids cannot be synthesized in mammals. Thus, the brain's elevated L‐phenylalanine, L‐lysine, and L‐isoleucine come either from the enhanced infusion or compromised decomposition. In our study, critical transporters of the XFZYD changed amine metabolites were not altered, which indicated that the elevated essential amino acids might not be due to enhanced transport. Thus, we speculated it to be caused by increased vasculature and blood supply in the brain. Angiogenesis is the process of new vessels sprouting from preexisting vasculatures. The enhanced brain vasculature can assist oxygen and metabolite exchange and remove necrotic debris.^61^ In addition, it cooperates with neurogenesis to promote brain recovery.^62^, ^63^ Previous studies demonstrated that microvascular density is compromised acutely after TBI.^63^ Then, the cerebral capillaries slowly reestablished to baseline levels within 2 weeks.^64^, ^65^ These are in correspondence with our results, showing no significant differences in vessel numbers and proliferating vessels between the sham‐operated and the TBI rats. However, 2 weeks after TBI, the hypoperfusion is still present,^66^ which explains the unrecovered histological and metabolic abnormalities in different regions of TBI brains on day 14. Thus, the natural process of angiogenesis was insufficient to fulfill TBI recovery.^62^, ^66^ We suggested that XFZYD ameliorates amine–metabolite abnormality by boosting angiogenesis and promoting the blood–brain exchange of essential amino acids and degrading products.

The pro‐angiogenic effect of XFZYD has been reported in many other diseases, such as myocardial infarction. The mechanism involves regulating the bFGF and VEGF/VEGFR2 signaling pathways.^67^, ^68^ In addition, many active compounds of XFZYD have been reported to promote angiogenesis. For example, hydroxysafflor yellow A in XFZYD can activate VEGF and Angiopoietin 1/Tie‐2 signaling pathway to promote angiogenesis.^69^, ^70^ Furthermore, ligustilide, ferulic acid, and 3‐n‐butylphthalide improve post‐stroke neurological deficits by facilitating angiogenesis.^71^, ^72^ Thus, the pro‐angiogenic effect of XFZYD on TBI brains may be an integrative result of the active compounds. In our study, XFZYD‐facilitated angiogenesis was more evident in the HP and TH than in other brain regions. It was agreed with the degree of region‐specific histological injury and metabolic alterations after TBI. We assumed that the regional variations of angiogenesis were caused by the differences in penetration rates of the active compounds through the blood–brain barrier.

### 4.5 Limitations

There are several limitations of the present study. First, to detect more metabolites, we select a relatively large extraction area in amine metabolomics at the expense of spatial resolution. In future work, a single‐cell‐resolution and wide‐coverage metabolomic method should be developed to explore the cell‐type‐specific metabolic alterations in different brain regions. Secondly, based on the published research, we discussed several potential amine–metabolites histological relations. However, the biological function of many differential metabolites and their correlations to brain histology are largely unknown. More studies are necessary to uncover it.

## 5 CONCLUSION

Taken together, this quantitative isomer‐differential spatial metabolomics and histological work reveals region‐specific amine metabolite and histopathological alteration after severe TBI at the subacute stage. In addition, XFZYD partially reverses the metabolic abnormalities of the TBI rat brain in a localized manner. These observations help to uncover the regional effect of different amine metabolites on other pathological processes of TBI and provide new evidence for the region‐specific effects of XFZYD on TBI.

## AUTHOR CONTRIBUTIONS

En Hu: investigation, resources, funding acquisition, validation, visualization, and writing – original draft; Tao Tang: data curation, methodology, funding acquisition, software, supervision, and writing – review & editing; You‐mei Li: investigation and validation; Teng Li: investigation, visualization, and writing – review & editing; Lin Zhu: investigation, resources, funding acquisition; Ruo‐qi Ding: investigation; Yao Wu: investigation; Qing Huang: formal analysis; Wei Zhang: formal analysis; Qian Wu: conceptualization, data curation, methodology, funding acquisition, software, supervision, and writing – review &editing; Yang Wang: conceptualization, data curation, funding acquisition, software, supervision, and writing – review & editing.

## CONFLICT OF INTEREST STATEMENT

The authors declare no competing financial interest.

## Supporting information


Appendix S1

Figure S4‐S5

## Data Availability

Data will be made available on request.
