# Supplementary material for: Spatial amine metabolomics and histopathology reveal localized brain alterations in subacute traumatic brain injury and the underlying mechanism of herbal treatment
Source: CNS Neurosci Ther. 2023 May 14;30(3):e14231. doi: 10.1111/cns.14231 (PMC10915989; doi:10.1111/cns.14231)
Supplement: Supplementary file 1 — Appendix S1 [file CNS-30-e14231-s002.docx]

**Supplementary material and method**

**1.1 Preparation and storage of XFZYD**

XFZYD is consist of 11 herbs: Prunus persica (L.) Batsch (Taoren), Carthamus tinctorius L. (Honghua), Angelica sinensis (Oliv.) Diels (Danggui), Rehmannia glutinosa (Gaertn.) DC. (Dihuang), Achyranthes bidentata Blume. (Niuxi), Citrus × aurantium L. (Zhiqiao), Paeonia lactiflora Pall. (Chisha), Glycyrrhiza uralensis Fisch. (Gancao), Ligusticum striatum DC. (Chuanxiong), Platycodon grandiflorus (Jacq.) A. DC. (Jiegeng), Bupleurum chinense DC. (Chaihu). The ratios of herbs were determined according to “TCM Prescriptions Dictionary”, plant name has been checked with <http://www.theplantlist.org> (Supplementary table 1). The herbs were purchased from Hunan Zhenxing Chinese Medicine Co., Ltd (Hunan, China. Drug GMP certificate: HN20150147. Drug Manufacturing Certificate: NO.20150021), and authenticated by Professor Suiyu Hu (Department of Chinese herbal medicine, Central South University, Changsha, China). Then, mixed herbs were extracted with double distilled water and vacuum-lyophilized to powder (yield = 16.9%, w/w), as we previously reported (Li et al. 2021, Li et al. 2020). To ensure the consistency of the chemical ingredients, the lyophilized powder was partitioned and sealed in a vacuum bag, and stored at -80℃. Working solution was made at a final concentration of 0.095 g/mL shortly before use. The concentrations of four major components were quantified by LC-MS (Supplementary Figure 1 and Supplementary table 2).

**1.2. In-situ spatial resolved sampling and amine labeling**

An LMJSS system was employed for spatial-resolved sampling based on our previous work (Wu et al. 2020). The flow probe was composed of two coaxial capillaries (outer capillary: 250 μm I.D./350 μm O.D.; inner capillary 100 μm I.D./170 μm O.D., Polymicro Technologies, Phoenix, AZ). The mixed solution of 10% hexafluoroisopropanol - 40% methanol-0.5% acetic acid-water was used as the extraction solvent. The extraction solvent was delivered through the outer capillary of the flow probe at a flowrate of 10 μL/min by a syringe pump (Longer Co., Baoding, China), and aspirated through the inner capillary by a diaphragm vacuum pump (MPC 301Z, WELCH, Mt. prospect, IL, USA). Liquid micro junction was formed on the probe-sample interface. Then the extracts were collected by an injection loop (11 μL). Meantime, the stability of LMJ was monitored by a microscopic camera (Zhong Xun Optical Instrument Co, Shenzhen, China).

The flow probe was positioned with x-y-z three linear stages (N-XYZ-ECRG-402020, Chengfang Optical Instrument Co. Shanghai, China), and controlled by a moving controller (KZQ03-300, Chengfang Optical Instrument Co. Shanghai, China). For spatial sampling, the flow probe was moved to the corresponding region (including the HP, the TH, the caudate-putamen (CP), the fiber tracts (FT), and the prefrontal CTX distal to the wound) and the regions were scanned by the probe spot-by-spot (6 spots per region).

The extracts collected from six spots of each region were differentially isotopically labeled with 6-plexed TMT6 reagents and then pooled together. For amino acid quantification, 3 μL of 22 amino acids (5 μg/mL) were labeled with TMT0 and then mixed with TMT6-labeled brain extracts to form one solution for LC-MS analysis. Standards labeled with TMT0 were used to calibrate the signals of the corresponding amino acids labeled with TMT6.

- 1. **Histological examination**

Paraffin sections were firstly deparaffined and rehydrated. For H&E staining, the rehydrated slices were stained with hematoxylin solution (G1004, Servicebio, Wuhan, China) for 5 min and eosin solution (G1001, Servicebio) for 20 sec. While Nissl staining was proceeded by immersing the sections into toluidine blue solution (G1036, Servicebio) for 8 min. Then, post-stained slices were dehydrated, hyalinized, and mounted for scanning. For immunofluorescence, the slices were incubated with the following primary antibodies at 4℃ overnight: mouse anti-GFAP (SC33673, 1:1500, Santa Cruz Biotechnology, Santa Cruz, CA), rabbit anti-Iba-1 (019-19741, 1:400, Wako Pure Chemical Industrials, Ltd., Osaka, Japan), rabbit anti-DCX (4604S, 1:800, Cell Signaling Technology, Beverly, MA, USA), rabbit anti-GAP43(8945S, 1:800, Cell Signaling Technology), rabbit anti-BDNF(ab108319, 1:1000, abcam, Cambridge, MA, USA), mouse anti-PCNA (SC-56, 1:1000, Santa Cruz Biotechnology) and vWF (A0082 1:400, Dako, Glostrup, Denmark). The corresponding Cy3 or Alexa Fluor^@^ 488-conjugated secondary antibodies were subsequently added for 1-hour incubation at room temperature. Afterward, the DAPI solution (C0065, Solarbio, Beijing, China) was stained for nuclear visualization. Finally, mounted sections were scanned using a Pannoramic Midi Scanner (3D Histech, Budapest, Hungary) and processed with the Caseviewer 2.3 (3D Histech).

- 1. **Molecular docking**

The 3D structures of tyrosine, Lys-Phe, and Phe-Lys were acquired from ZNIC15 (http://zinc.docking.org/; ZINC266964; ZINC2522657; ZINC13520785). The crystal structures of tyrosine hydroxylase were obtained from the RCSB Protein Data Bank (https://www.rcsb.org/, identifier: 1TOH). Small molecules and targets were converted to pdbqt formats with AutoDockTools 1.5.6. The water was deleted and polar hydrogen atoms were added to the protein. Then, the molecular docking was performed utilizing the AutoDock Tools package (version 1.5.6) and Autodock Vina (version 1.1.2). The active pocket of target was coordinated at center_x = 71.645, size_x = 64, center_y = 56.829, size_y = 54, center_z = 36.878, size_z = 80. The exhaustiveness was set to 50. The docking process was calculated by the Genetic Algorithm. Other parameters were set as default. Finally, the molecular conformations of the ligands with the highest affinity to the targets were visualized by PyMoL.The docking process was calculated by the Genetic Algorithm. Other parameters were set as default. Finally, the molecular conformations of the ligands with the highest affinity to the targets were visualized by PyMoL.The docking process was calculated by the Genetic Algorithm. Other parameters were set as default. Finally, the molecular conformations of the ligands with the highest affinity to the targets were visualized by PyMoL.

1. **Supplementary tables**

**2.1 Supplementary Table 1. Composition XFZYD.**

| **Chinese name** | **Plant name** | **Latin name** | **Part used** | **Origin Area** | **Batch number** | **Ratios** |
| --- | --- | --- | --- | --- | --- | --- |
| Taoren | *Prunus persica* (L.) Batsch | *Semen Persicae* | Seed | Hebei | 19061010 | 8 |
| Honghua | *Carthamus tinctorius* L. | *Flos Carthami* | Flower | Xinjiang | 19080108 | 6 |
| Danggui | *Angelica sinensis* (Oliv.) Diels | *Radix Angelicae Sinensis* | Root | Gansu | 20190902 | 6 |
| Dihuang | *Rehmannia glutinos*a (Gaertn.) DC. | *Radix Rehmanniae* | Root | Henan | 19072305 | 6 |
| Niuxi | *Achyranthes bidentata* Blume. | *Radix Achyranthis Bidentatae* | Root | Sichuan | 19041505 | 6 |
| Zhiqiao | *Citrus × aurantium* L. | *Fructus Aurantii* | Fruit | Jiangxi | 19051003 | 4 |
| Chishao | *Paeonia lactiflora* Pall. | *Radix Paeoniae Rubra* | Root | Sichuan | 19062607 | 4 |
| Gancao | *Glycyrrhiza uralensis* Fisch. | *Radix Glycyrrhizae* | Root | Inner Mongolia | 19041313 | 4 |
| Chuanxiong | *Ligusticum striatum* DC. | *Rhizoma Chuanxiong* | Root | Sichuan | 19062904 | 3 |
| Jiegeng | *Platycodon grandiflorus* (Jacq.) A. DC. | *Radix Platycodonis* | Root | Sichuan | 19061512 | 3 |
| Chaihu | *Bupleurum chinense* DC. | *Radix Bupleuri* | Root | Hebei | 19052910 | 2 |

**2.2 Supplementary Table 2.** **Concentrations of active ingredients in XFZYD solution**.

| **Metabolites** | **Origin** | **Exact Mass** | **Q1 mass (Da)** | **Q3 mass (Da)** | **Retention time (min)** | **Adduct** | **Concentrations**  **(μg/mL)** |
| --- | --- | --- | --- | --- | --- | --- | --- |
| Hydroxysafflor yellow A | Carthami flos | 612.16903493 | 611.200 | 491.100 | 1.25 | [M-H]^-^ | 3882.00±63.008^a^ |
| Ferulic acid | Angelicae sinensis radix and Chuanxiong rhizome | 194.05790880 | 192.900 | 133.900 | 3.30 | [M-H]^-^ | 9.57±0.084^a^ |
| paeoniflorin | Paeoniae radix rubra | 480.16316171 | 479.200 | 449.100 | 2.21 | [M-H]^-^ | 66.24±0.934^a^ |
| amygdalin | Persicae semen | 457.15841068 | 456.100 | 323.100 | 1.49 | [M-H]^-^ | 49.30±0.757^a^ |

^a^ Data are expressed as mean ± SD, n = 5.

**2.3 Supplementary table 3.** the detected amino acids and differentially expressed amine metabolites by LMJSS–TMT–LC–MS analysis on brain tissues

| **Metabolites** | **Theoretical MW** | ***m/z* after TMT6 derivation** | **Predicted *m/z* after TMT6 derivation** | **Retention time （min）** | **Adduct ions** | **△ppm^a^** | **Fragment^b^** | **HMDB ID** | **Significance**  **(CCI *vs* Sham/XFZYD *vs* CCI)** | | | | |
| --- | --- | --- | --- | --- | --- | --- | --- | --- | --- | --- | --- | --- | --- |
|  |  |  |  |  |  |  |  |  | **HP** | **TH** | **CP** | **FT** | **CTX** |
| L-isoleucine | 131.0946 | 361.2530 | 361.2544 | 11.69 | M+H | 4 | 126.1-131.1 | HMDB0000172 | ns/ns | ns/ns | ↓**/  #↑ | ↓*/ns | ns/ns |
| L-leucine | 131.0946 | 361.2530 | 361.2544 | 12.17 | M+H | 4 | 126.1-131.1 | HMDB0000687 | ns/ns | ns/ns | ↓**/ns | ↓*/#↑ | ↓**/ns |
| β-alanine | 89.0476 | 319.2075 | 319.2074 | 2.36 | M+H | 1 | 126.1-131.1 | HMDB0000056 | ns/ns | ↑*/ns | ↑*/ns | ↑*/ns | ↑*/ns |
| α-alanine | 89.0476 | 319.2075 | 319.2074 | 1.97 | M+H | 1 | 126.1-131.1 | HMDB0000161 | ns/ns | ns/ns | ns/ns | ns/ns | ns/ns |
| L-glutamine | 146.0691 | 376.2272 | 376.2289 | 1.28 | M+H | 5 | 126.1-131.1 | HMDB0000641 | ns/ns | ns/ns | ns/ns | ns/ns | ns/ns |
| L-tyrosine | 181.0738 | 411.2336 | 411.2336 | 7.63 | M+H | 0 | 126.1-131.1 | HMDB0000158 | ns/ns | ns/ns | ns/ns | ns/ns | ns/ns |
| Norvaline | 117.0789 | 347.2380 | 347.2387 | 8.39 | M+H | 2 | 126.1-131.1 | HMDB0000883 | ns/ns | ns/ns | ns/ns | ns/ns | ns/ns |
| L-valine | 117.0789 | 347.2380 | 347.2387 | 7.54 | M+H | 2 | 126.1-131.1 | HMDB0000883 | ↓*/ns | ns/ns | ↓*/ns | ns/ns | ns/ns |
| γ-aminobutyric acid | 103.0633 | 333.2223 | 333.2230 | 2.60 | M+H | 2 | 126.1-131.1 | HMDB0000112 | ↓***/  ns | ↓*/ns | ns/ns | ns/ns | ns/ns |
| L-phenylanine | 165.0789 | 395.2380 | 395.2387 | 13.08 | M+H | 2 | 126.1-131.1 | HMDB0000159 | ns/ns | ns/ns | ↓*/#↑ | ns/ns | ns/ns |
| L-glutamic acid | 147.0531 | 377.2112 | 377.2129 | 1.41 | M+H | 5 | 126.1-131.1 | HMDB0000148 | ↓**/ns | ns/ns | ns/ns | ns/ns | ns/ns |
| L-serine | 105.0425 | 335.2011 | 335.2023 | 1.23 | M+H | 4 | 126.1-131.1 | HMDB0000187/ HMDB0003406 | ns/ns | ns/ns | ns/ns | ns/ns | ns/ns |
| L-lysine | 146.1055 | 303.2125 | 303.2125 | 5.83 | M+2H | 0 | 126.1-131.1 | HMDB0000182 | ns/ns | ns/ns | ns/ns | ↓*/ns | ↓*/#↑ |
| Thioproline/5-methyltetrahydrofolic acid | 133.0197/459.1866 | 345.1675 | 345.1703/345.1695 | 11.23 | M+H-H2O/M+2H | 8/6 | 126.1284, 225.1576, 243.1693, 312.1892 | HMDB0062164/HMDB0001396 | ↑**/ns | ns/ns | ns/ns | ns/ns | ns/ns |
| Lys-Phe/Phe-Lys | 293.1739 | 262.1547 | 262.1622 | 14.23 | M+2H | 29 | 126.1-131.1, 244.1493 | HMDB0028958/HMDB0029000 | ↑*/  ##↓ | ns/ns | ns/ns | ns/ns | ns/ns |
| Gly-Thr/Ser-Ala | 176.0797 | 406.2338 | 406.2321 | 11.20 | M+H | 4 | 183.1499, 225.1575, 243.1675 | HMDB0028851/HMDB0029032 | ↑*/ns | ns/ns | ns/ns | ns/ns | ns/ns |
| Arg-His | 311.1705 | 271.1559 | 271.1614 | 7.27 | M+2H | 20 | 126.1281 | HMDB0028711 | ↓*/ns | ns/ns | ns/ns | ns/ns | ns/ns |
| 5-hydroxylysine/4-amino-1-piperidinecarboxylic acid | 162.1004/144.0898 | 356.2511 | 356.2528/356.2422 | 12.19 | M+H-2H2O/M+H-H2O | 5/  25 | 126.1283, 127.133, 171.1482, 183.1498, 225.1575, 243.1667 | HMDB0000450/HMDB0060385 | ↑*/ns | ns/ns | ns/ns | ns/ns | ns/ns |
| Arg-Leu/Arg-Ile | 287.1957 | 259.1711 | 259.1740 | 6.87 | M+2H | 11 | 59.9693, 126.1-128.1 | HMDB0028713/HMDB0028712 | ns/ns | ↓*/ns | ns/ns | ns/ns | ns/ns |

^a^: △ppm = (Predicted m/z - m/z)/Predicted m/z * 10^^6^. b: fragments were from MS/MS spectra obtained in multiple stage fragmentation scan mode. * *P* < 0.05, ** *P* <0.01, *** *P* < 0.005 (CCI *vs* Sham); # *P* < 0.05, ## *P* < 0.01 (XFZYD vs CCI); ns: *P* > 0.05. ↑ upregulated, ↓ downregulated.

1. **Supplementary figures**

**Supplementary Figure 1.** LC chromatogram of hydroxysaffor yellow A, ferulic acid, paeoniflorin, and amygdalin in XFZYD. **
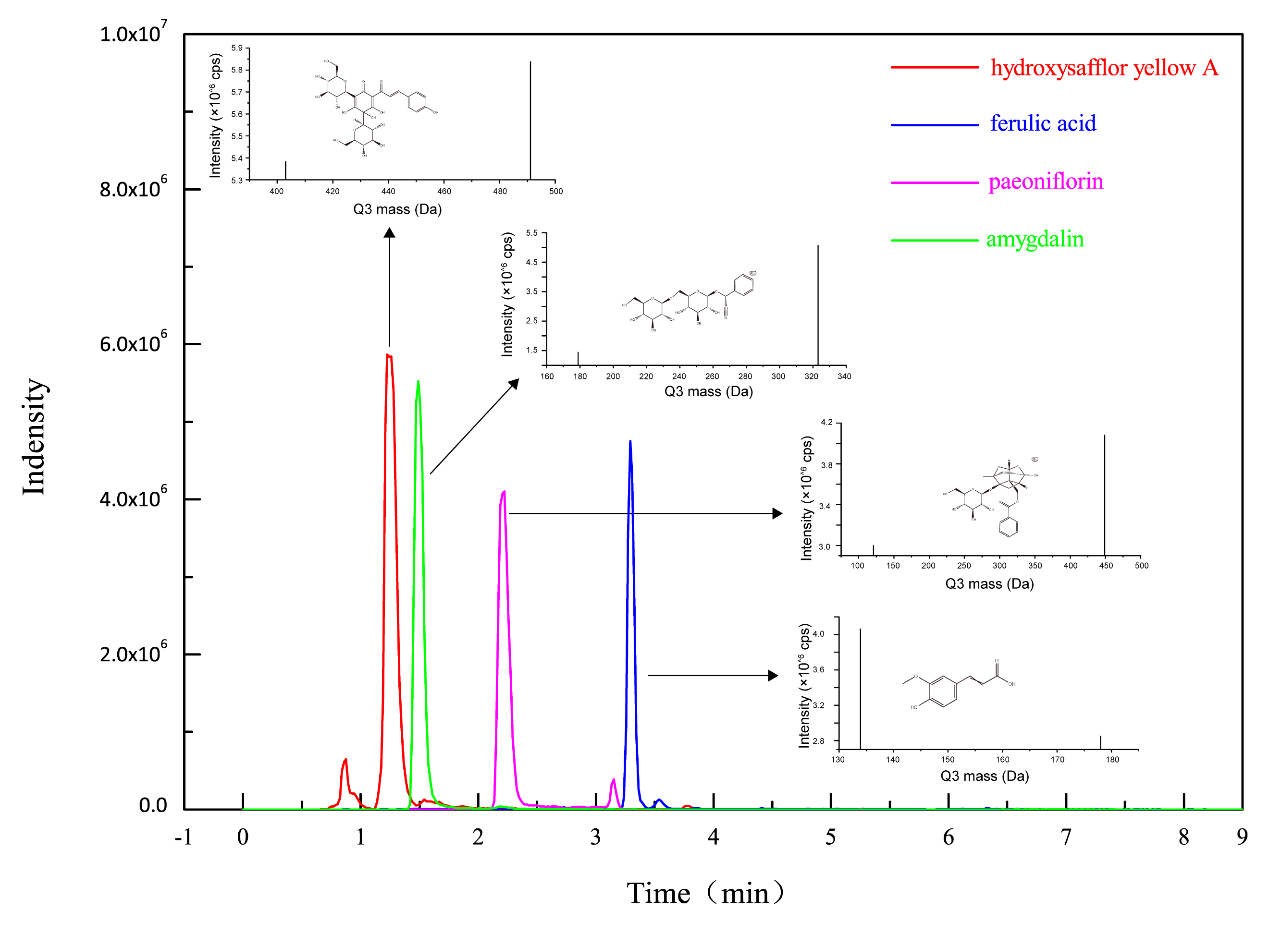
**

**
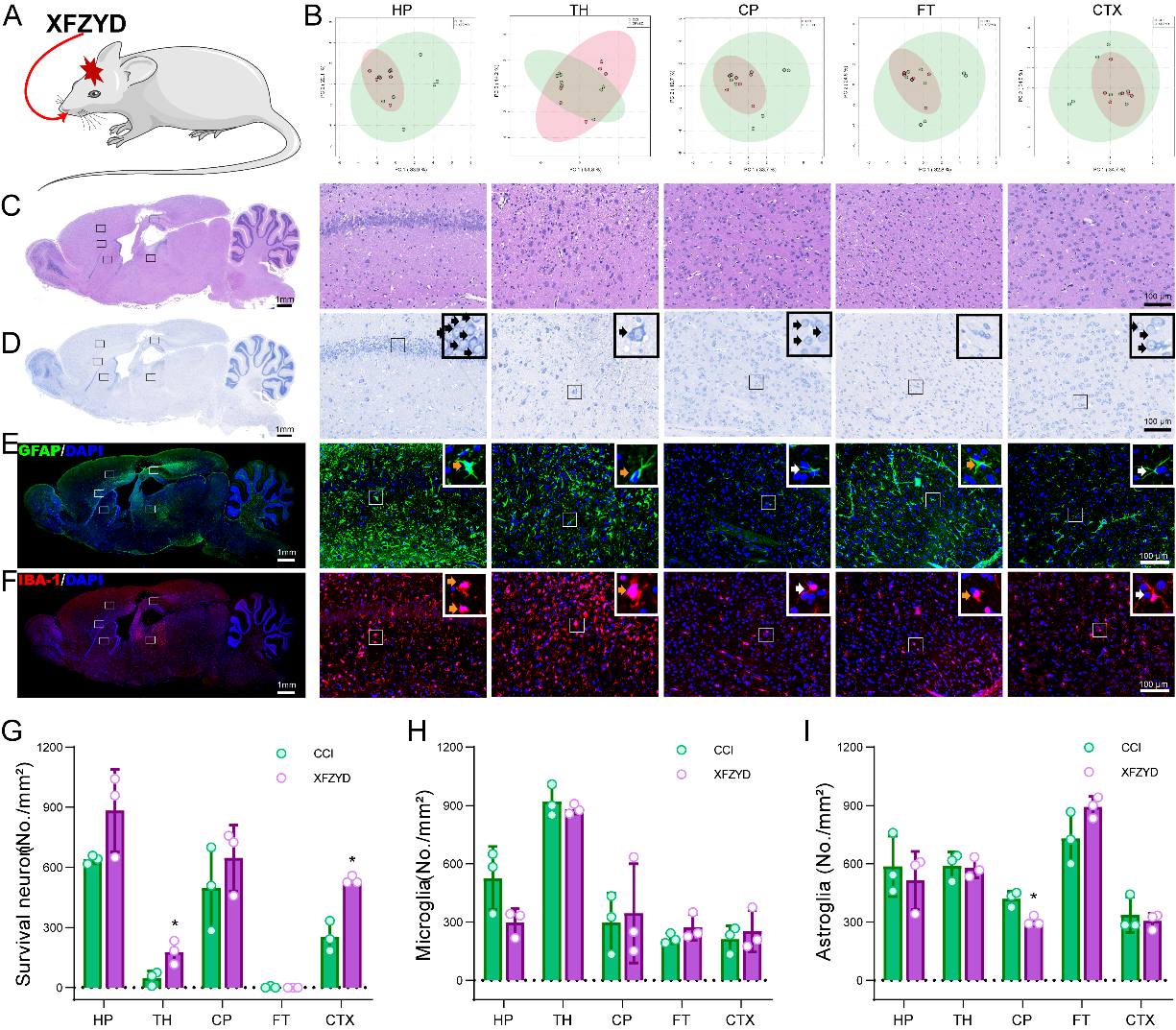
Supplymentary Figure 2.** **Amine metabolites and histopathological changed after XFZYD treatment.** (A) Rat were oral administrated with XFZYD. (B) PCA plots showed moderate differences in the overall profiles of amine metabolites between CCI group and XFZYD group in the CP, CTX, FT, HP, and TH. (C) H&E staining showed general histopathological changes after XFZYD treatment. (D) Nissl staining showed neuron damage after XFZYD treatment. (E) immunofluorescent showed microglia status after XFZYD treatment. (F) immunofluorescence showed astrocyte status after XFZYD treatment. (G) Statistic of Nissl staining indicated improved neuron loss after XFZYD treatment in the TH and the CTX. (H) Statistic of microglia staining indicated no significant change after XFZYD treatment in the 5 regions. (I) Statistic of astrocyte staining indicated decreased astrocytes number in the CP after XFZYD treatment. HP: hippocampus; TH: the thalamus; CP: the caudate-putamen; FT: the fiber tracts; CTX: the cortex. N = 6 in the CCI group, n = 8 in the XFZYD group (B); n = 3 (C-I) , * *P* < 0.05; ** *P* < 0.01.


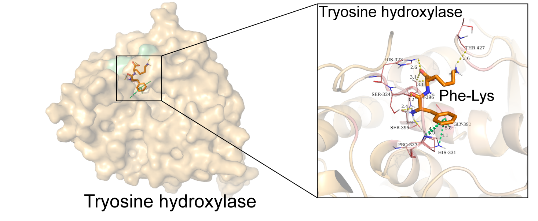


**Supplementary Figure 3.** **Molecular docking of Phe-Lys with tyrosine hydroxylase.** Yellow dotted lines: H-bond; green dotted lines: pi-cation bonds.
