# Supplementary material for: Spatial amine metabolomics and histopathology reveal localized brain alterations in subacute traumatic brain injury and the underlying mechanism of herbal treatment
Source: CNS Neurosci Ther. 2023 May 14;30(3):e14231. doi: 10.1111/cns.14231 (PMC10915989; doi:10.1111/cns.14231)
Supplement: Supplementary file 2 — Figure S4‐S5 [file CNS-30-e14231-s001.pdf]

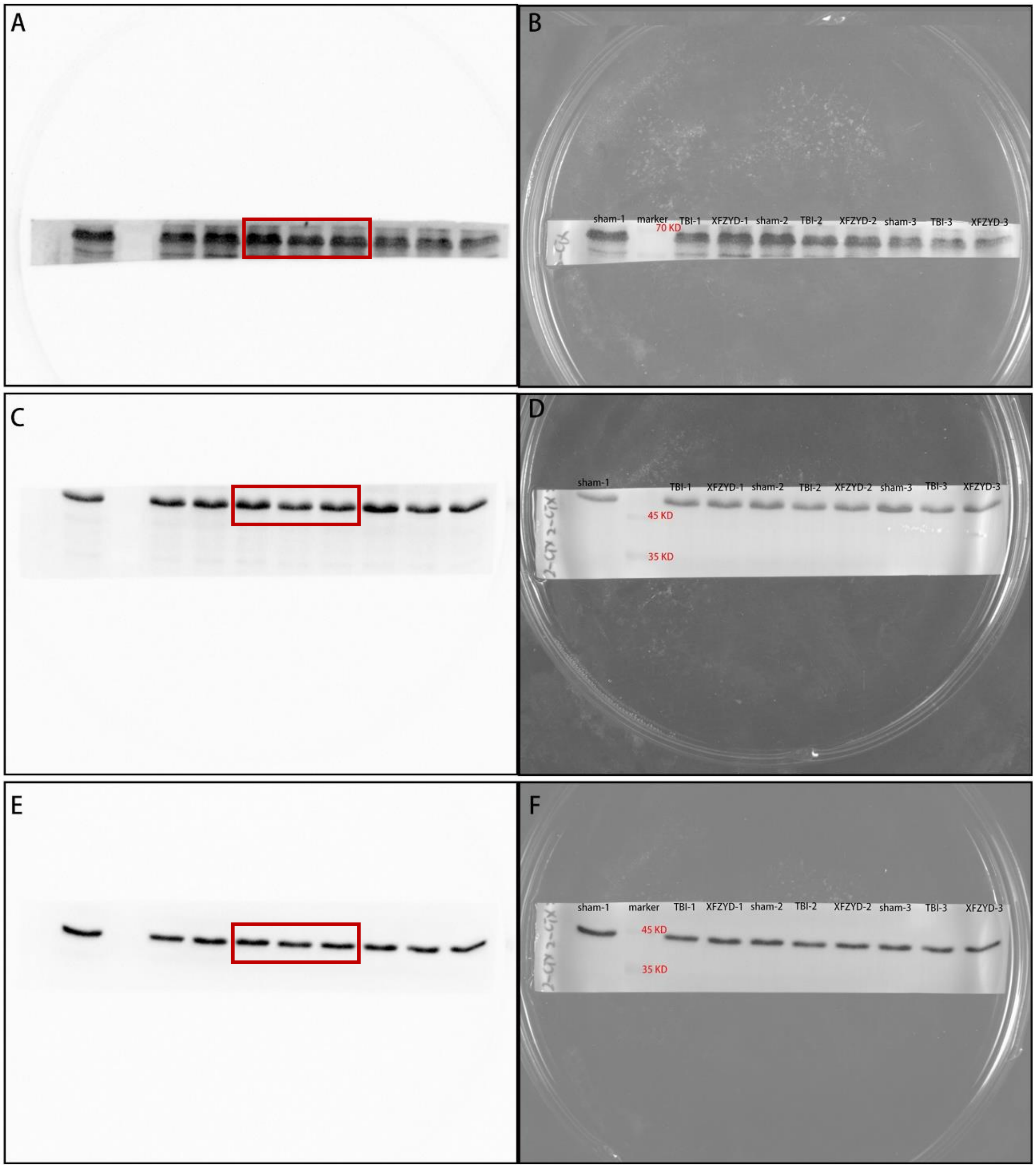

Supplementary Figure 1. The original, uncropped image in CTX. A. The uncropped image of Slc3a2 in CTX for Figure 6G. B. The merged blot shows the location of molecular weight standards corresponding to Slc3a2 in CTX for Figure 6G. C. The uncropped image of Slc7a5 in CTX for Figure 6G. D. The merged blot shows the location of molecular weight standards corresponding to Slc7a5 in CTX for Figure 6G. E. The uncropped image of β-actin in CTX for Figure 6G. F. The merged blot shows the location of molecular weight standards corresponding to β-actin in CTX for Figure 6G. The cropped images used in the manuscript are marked in red rectangles.

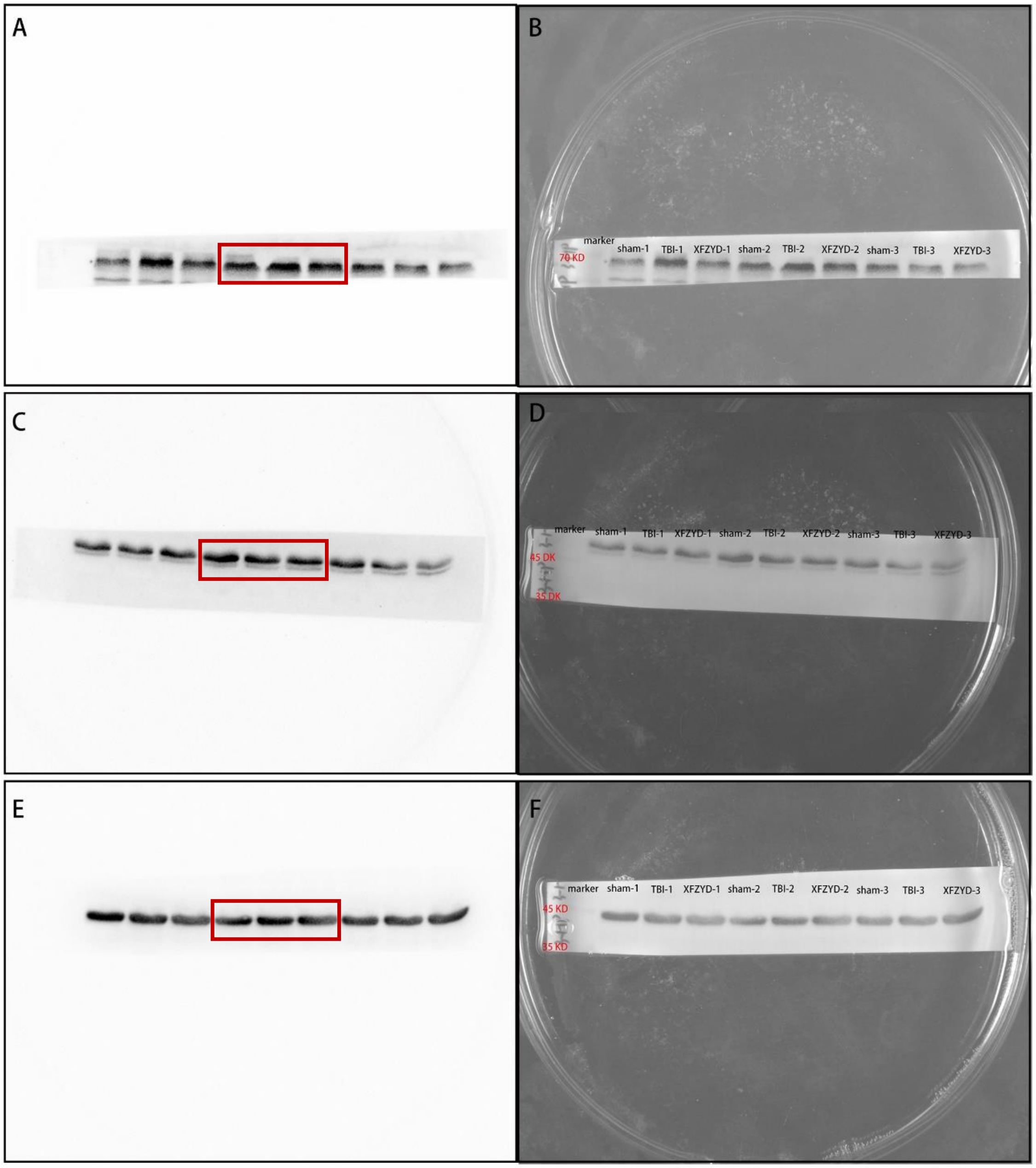

Supplementary Figure 2. The original, uncropped image in HP. A. The uncropped image of Slc3a2 in HP for Figure 6G. B. The merged blot shows the location of molecular weight standards corresponding to Slc3a2 in HP for Figure 6G. C. The uncropped image of Slc7a5 in HP for Figure 6G. D. The merged blot shows the location of molecular weight standards corresponding to Slc7a5 in HP for Figure 6G. E. The uncropped image of β-actin in HP for Figure 6G. F. The merged blot shows the location of molecular weight standards corresponding to β-actin in HP for Figure 6G. The cropped images used in the manuscript are marked in red rectangles for Figure 6G.
